# Traditional use of medicinal plants in south-central Zimbabwe: review and perspectives

**DOI:** 10.1186/1746-4269-9-31

**Published:** 2013-05-04

**Authors:** Alfred Maroyi

**Affiliations:** 1 Department of Biodiversity, School of Molecular and Life Sciences, University of Limpopo, Mankweng, South Africa

**Keywords:** Conservation, Medicinal plants, South-central Zimbabwe, Traditional knowledge

## Abstract

**Background:**

Traditional medicine has remained as the most affordable and easily accessible source of treatment in the primary healthcare system of resource poor communities in Zimbabwe. The local people have a long history of traditional plant usage for medicinal purposes. Despite the increasing acceptance of traditional medicine in Zimbabwe, this rich indigenous knowledge is not adequately documented. Documentation of plants used as traditional medicines is needed so that the knowledge can be preserved and the utilized plants conserved and used sustainably. The primary objective of this paper is to summarize information on traditional uses of medicinal plants in south-central Zimbabwe, identifying research gaps and suggesting perspectives for future research.

**Methods:**

This study is based on a review of the literature published in scientific journals, books, reports from national, regional and international organizations, theses, conference papers and other grey materials.

**Results:**

A total of 93 medicinal plant species representing 41 families and 77 genera are used in south-central Zimbabwe. These plant species are used to treat 18 diseases and disorder categories, with the highest number of species used for gastro-intestinal disorders, followed by sexually transmitted infections, cold, cough and sore throat and gynaecological problems. Shrubs and trees (38% each) were the primary sources of medicinal plants, followed by herbs (21%) and climbers (3%). The therapeutic claims made on medicinal plants documented in south-central Zimbabwe are well supported by literature, with 82.8% of the plant species having similar applications in other regions of Zimbabwe as well as other parts of the world and 89.2% having documented biological and pharmacological properties.

**Conclusion:**

This study illustrates the importance of traditional medicines in the treatment and management of human diseases and ailments in south-central Zimbabwe. Traditional medicines still play an important role in meeting basic health care of local communities in Zimbabwe.

## Background

**Figure 1:**
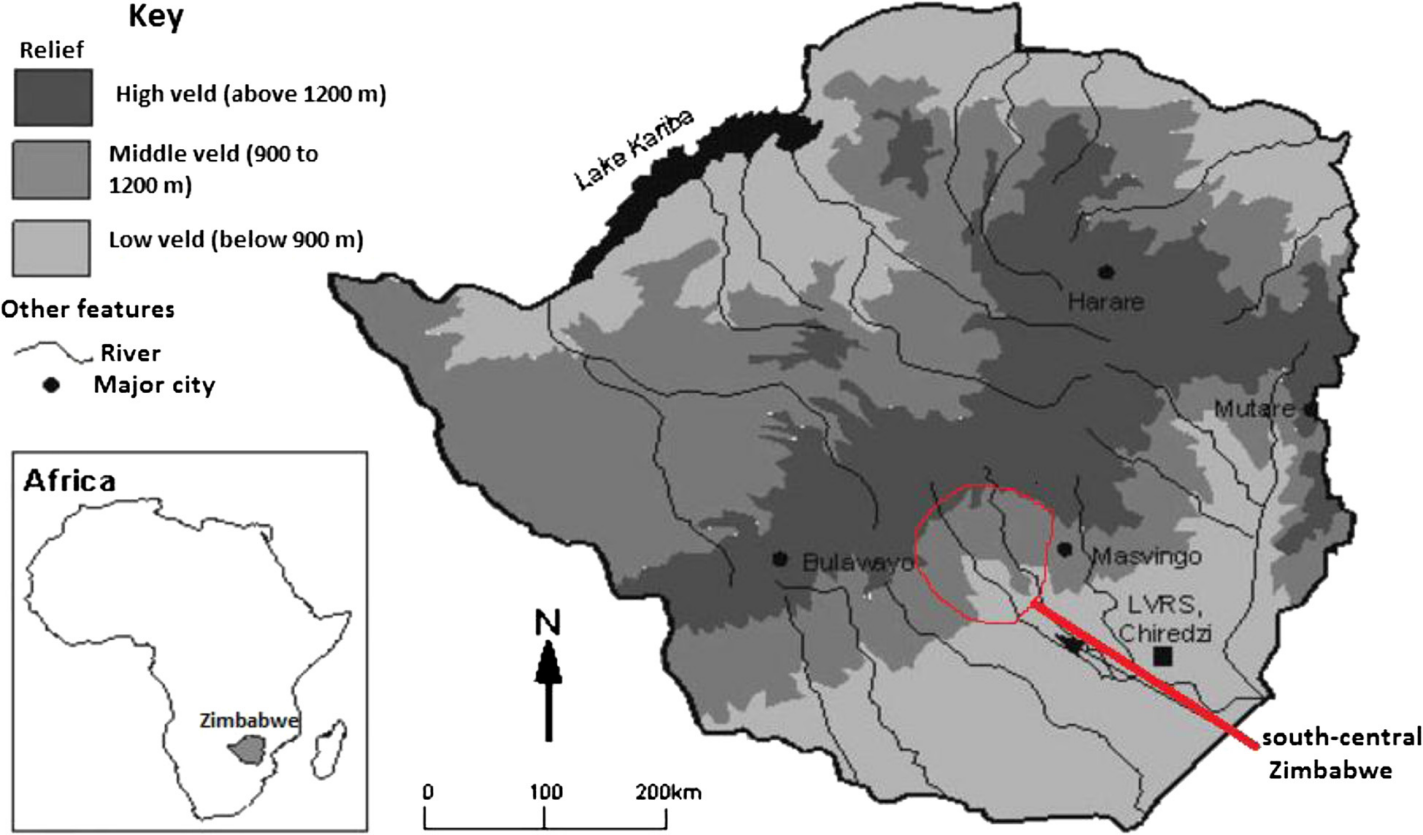
**Geographical location of the study area, map modified from**[6]**.**

Out of more than 5000 plant species growing in Zimbabwe, about 10 percent of these have medicinal properties and are used as traditional medicines [1]. Traditional medicine has remained as the most affordable and easily accessible source of treatment in the primary healthcare system of resource poor communities in Zimbabwe. About 80% of the population in developing countries use traditional medicines because they cannot afford the high cost of western pharmaceuticals and health care, and because traditional medicines are more acceptable from a cultural and spiritual perspective [2]. Research by Hostettmann et al. [3] showed that the knowledge on the use of medicinal plants is enormous but if this traditional knowledge is not rapidly researched and recorded, indications are that it will be lost with succeeding generations. Despite the increasing acceptance of traditional medicine in Zimbabwe [1,4,5], this rich indigenous knowledge on traditional remedies is not adequately documented. Documentation of plants used as traditional medicines in Zimbabwe is urgent so that the knowledge can be preserved, the utilized plants are conserved and used sustainably. The current investigation therefore, attempts to fill some of the gaps in indigenous knowledge related to the use of herbal medicines in south-central Zimbabwe (Figure 1) emphasizing their role in basic human health care. The majority of the people in south-central Zimbabwe belong to the Karanga dialectical group. Most of the Karanga people live in Mberengwa, Shurugwi and Zvishavane districts in the Midlands province; and Chivi and Zaka districts in the Masvingo province [7], which are the study sites of the current study. The Karanga people possess their own traditional knowledge on medicinal plants that contributes to a broader understanding of medicinal plants in Zimbabwe. This investigation is part of a larger study [8] aimed at documenting the ethnobotanical knowledge held by the Karanga people in south-central Zimbabwe. Therefore, this review was done to document traditional uses of medicinal plants in south-central Zimbabwe in order to provide comprehensive documentation, identify research gaps, and suggest perspectives for future research.

## Methods

The traditional uses of medicinal plants in south-central Zimbabwe were collated. Available references or reports on the plant species were consulted from published scientific journals, books, reports from national, regional and international organizations, theses, conference papers and other grey materials. Literature was searched on international online databases such as ISI Web of Science, MEDLINE, Science Direct, Scopus and Google Scholar using specific search terms such as “medicinal plants”, “traditional medicines”, “Chivi or Mberengwa or Shurugwi or Zaka or Zvishavane districts”, “Masvingo or Midlands provinces” and “Zimbabwe”. References were also identified by searching the library collections of the National Herbarium and Botanic Gardens, Harare, Zimbabwe and University of Limpopo, South Africa. Data collected from the literature included: use(s), mode of preparation and administration of the species. Literature search was also done to document the biological and pharmacological activities of the documented plant species.

### Medicinal plant diversity

This study recorded ninety three plant species as useful in traditionally managing various human diseases in south-central Zimbabwe (Table 1). Of these, 79 species are indigenous to Zimbabwe (84.9%), while 14 species are exotic (15.1%), either naturalized as weeds or cultivated in home gardens as ornamentals or food plants. Dicotyledons were dominant with 87 plant species (93.5%), 5 monocotyledons (5.4%) and 1 fern (1.1%). These medicinal plants were distributed among 41 families and 77 genera. The majority of medicinal plants (71, 76.3%) used in south-central Zimbabwe are from 19 families (Table 2). Plant families with the highest number of medicinal plants in south-central Zimbabwe were: Fabaceae *sensu lato* (11 species), followed by Anacardiaceae (9 species), Euphorbiaceae (7 species), Asteraceae, Ebenaceae and Tiliaceae (4 species each). Fabaceae, Anacardiaceae and Euphorbiaceae families have the highest number of species used as herbal medicines probably because these are large families characterized by several species. The rest of the families were represented by one species each (Table 1). The genera with highest number of species were *Ficus, Grewia, Searsia* and *Strychnos* with 3 species each.

**Table 1:** Medicinal plants used in south-central Zimbabwe

**Scientific name, family**	**Growth habit**	**Vernacular name**	**Part(s) used and use(s)**	**Similar uses in Zimbabwe (other countries**^**#**^**) reported from literature**	**Reported biological/pharmacological activities**
**Anacardiaceae**					
*Lannea discolor* (Sond.) Engl.	Tree	Mugan’acha	**Fibre:** extract drunk to reduce the duration of menstrual flow [9]. **Roots:** root extract used as eye drops for sore eyes [8]	Menorrhagia [1] and infertility^#^[1,10]	None found
*Lannea edulis* (Sond.) Engl.	Shrub	Mutsambatsi	**Roots:** extract drunk as bilharzia (schistomiasis) and diarrhoea [9] and gonorrhoea medicine [8]	Gonorrhoea [1] and bilharzias^#^, diarrhoea^#^[11]	Antimicrobial activity; flavonoids and tannins [12]
*Mangifera indica L.	Tree	Mumango	**Bark:** extract drunk as diarrhoea medicine [8]	Diarrhoea and dysentery [13]	Antibacterial [13,14]; anti-inflammatory, antifungal, antidiabetic, antioxidant, antiviral and antiparasitic [14] properties
*Ozoroa insignis* Del.	Shrub	Mubhedha	**Roots:** extract drunk as diarrhoea and STIs medicine [8]	Diarrhoea and venereal diseases [1]	Antibacterial, anthelmintic [15,16], antimicrobial [16] and cytotoxic [17] activities
*Rhus longipes* Engl.	Tree	Mufokosiana	**Roots:** extract drunk as remedy for infertility in women and to dilate birth canal [8]	Infertility in women and to dilate birth canal [1]	None found
*Sclerocarya birrea* (A. Rich.) Hochst.	Tree	Mupfura	**Roots:** steam directed into sore eyes [8]	Sore eyes [1]	Flavonoids, tannins and triterpenoids; antidiarrhoeal, antidiabetic, anti-inflammatory, antimicrobial and antioxidant [18] properties
*Searsia dentata* (Thunb.) F.A. Barkley	Shrub	Mubikasadza	**Leaves:** leaf sap taken as remedy for ulcers, diarrhoea and stomach problems [9]	None found	Biflavonoids [19]
*Searsia pyroides* (Burch.) Moffett	Shrub	Mufokosiana	**Roots:** extract drunk as cough medicine [8]	None found	Biflavonoids [20]
*Searsia tenuinervis* (Engl.) Moffet	Shrub	Mufokosiana	**Leaves:** extract drunk as menorrhagia medicine [8]	Menorrhagia [1]	Antibacterial activity [21]
**Annonaceae**					
*Annona stenophylla* Engl. & Diels	Shrub	Muroro	**Roots:** paste applied on the boils; extract drunk as chest pains and STI remedy; mixed with roots of *Securidaca longipedunculata* Fresen. and sprinkled around homestead as snake repellent [8]	Gonorrhoea, syphilis and snake repellent [1]	Antioxidant activity [22]
**Apocynaceae**					
*Carissa bispinosa* (L.) Desf. ex Brenan	Shrub	Muruguru	**Roots:** extract drunk as cough and diarrhoea medicine [8]	None found	Analgesic, antiviral and diuretic activities; lignans and sesquiterpenes [23]
*Carisa edulis* (Forssk.) Vahl	Shrub	Muruguru	**Roots:** extract drunk as diarrhoea and cough medicine [6]	Cough [1,24], chest pains and pneumonia [1]; and tuberculosis^#^[10]	Analgesic, antiviral and diuretic activities; lignans and sesquiterpenes [23]
*Catharanthus roseus (L.) G. Don	Herb	Chirindamatongo	**Roots:** extract drunk as remedy for stomach problems [9,25]	Diarrhoea^#^, dysentery^#^ and indigestion^#^[26]	Alkaloids, flavonoids, saponins, tannins and triterpenes; antidiarrheal [27] and antidiabetic [28] activities
**Asparagaceae**					
*Asparagus africanus* Lam.	Climber	Rukato	**Roots:** extract drunk as diarrhoea and pneumonia medicine and to dilate birth canal [8]	Aid in child birth^#^[29]	Analgesic and anti-inflammatory activities [30]
*Sansevieria aethiopica* Thunb.	Herb	Zvikonje	**Leaves:** leaf sap squeezed into the painful ear [31]	Earache^#^[32]	Antibacterial activity [33]
*Sansevieria hyacinthoides* (L.) Druce	Herb	Masavamhanda	**Leaves or rhizomes:** leaf or rhizome sap given to child suffering from dehydration [31]. **Leaves:** Leaf macerate given to colicky infant; leaves used as dressing on sprained ankle [31]. **Rhizome:** rhizome added to non-alcoholic beverage taken by pregnant women to prepare the birth canal and prevent delivery complications; warm rhizome extract given to a person with aching tooth to keep in mouth for up to two minutes before spitting out the mixture [31].** Roots:** extract drunk to dilate birth canal [9]	Root used as baby food^#^[10]	Antibacterial, antioxidant [34] and anti-inflammatory [12] properties.
**Asphodelaceae**					
*Aloe greatheadii* Schönland	Herb	Gavakava	**Leaves:** extract drunk as constipation and gonorrhoea medicine [8]	Constipation and gonorrhoea [1]	Alkaloids, phenolic compounds and antioxidant capacity [35]
**Asteraceae**					
*Brachylaena discolor* DC.	Herb	Mupasa	**Leaves:** leaves chewed and juice swallowed as remedy for ulcers [9]	Enema to stop bleeding of the stomach^#^[36]	Antidiabetic activity [28]
*Dicoma anomala* Sond.	Herb	Chifumuro	**Bulb:** extract drunk as remedy for stomach upset [9]	Remedy for all disease (panacea) [1]	Sesquiterpene [37]
*Schkuhria pinnata (Lam.) Kuntze ex Thell.	Herb	Ruhwahwa	**Whole plant:** extract drunk as remedy for stomach pains [9]	Stomach problems^#^[11]	Antibacterial [38] and anti-diarrhoeal activity [11]
*Sonchus oleraceus L.;	Herb	Rurimirwemombe	**Leaves:** extract drunk as remedy for stomach problems [9]	Anti-diarrhoeal^#^ and digestive purgative^#^[39]	Alkaloids, flavonoids, phenols and saponins; antioxidant and antibacterial [39] properties
**Burseraceae**					
*Commiphora marlothii* Engl.	Tree	Mupepe	**Roots:** extract drunk as STI medicine [9]	None found	Antibacterial activity [40]
**Celastraceae**					
*Gymnosporia buxifolia* (L.) Szyszyl.	Shrub	Chizhuzhu	**Leaves:** leaves chewed and sap swallowed as remedy for abdominal pains [9]	Painful menstruation^#^[41]	Antiplasmodial and anti-inflammatory activities [42]
**Chrysobalanaceae**					
*Parinari curatellifolia* Planch. ex Benth.	Tree	Muchakata	**Roots:** extract drunk as constipation medicine and teeth washed with root decoction as remedy for toothache [8]	Constipation [1] and toothache^#^[1,10]	Antibacterial [13,43] and antimicrobial [43] activities; alkaloids, flavonoids, phenol, saponins, steroids, tannins and terpenes [43]
**Clusiaceae**					
*Garcinia buchananii* Baker	Tree	Mutunduru	**Bark:** extract drunk to reduce birth canal [9]. **Fruits:** ripe fruits are eaten as aphrodisiac [9]	Aphrodisiac and to reduce birth canal [1]	Anthraquinones and cytotoxicity activity [44,45], phenolics, steroids and tannins [46]
**Cucurbitaceae**					
*Cucumis anguria* L.	Herb	Muchacha	**Fruits:** pieces of fruit left around homestead as snake repellent [8]	Antifeedant [47]	Larvicidal activity and triterpenoids [47]
**Cyperaceae**					
*Coleochloa setifera* (Ridl.) Gilly	Herb	Rufuri	**Roots:** root powder taken as a remedy for pneumonia [12]	None found	None found
**Ebenaceae**					
*Diospyros lycioides* Desf.	Shrub	Musumadombo	**Roots:** extract drunk as remedy for infertility in women [8]	Infertility in women^#^[1,10]	Antibacterial activity [48]; lupeol and ursolic acid [49]
*Diospyros mespiliformis* Hochst. ex A.DC.	Tree	Musuma	**Roots:** extract drunk as abdominal pains medicine [8]	Body and heart pains [1]	Antimicrobial activity; saponins, steroids, tannins and triterpene [50]
*Euclea crispa* (Thunb.) Sond. ex Gürke	Shrub	Muvhinji	**Roots:** extract drunk as cough medicine [8]	Cough [1]	Antibacterial activity [51]
*Euclea divinorum* Hiern	Shrub	Mushangura	**Roots:** extract drunk as diarrhoea medicine [8]	Diarrhoea [1]; and troubled and noisy stamach^#^[10]	Antimicrobial activity [44]
**Euphorbiaceae**					
*Androstachys johnsonii* Prain	Tree	Musimbiti	**Roots:** extract drunk as aphrodisiac [9]	None found	Antibacterial [52], antimicrobial and antifungal [53] activities
*Bridelia cathartica* G. Bertol.	Shrub	Mutsvoritsvoto	**Roots:** extract drunk as remedy for infertility in men [8]	Infertility in men [1]	Anthocyanins, flavonoids and tannins [54]; antibacterial [54] and antimalarial [55] activities
*Bridelia mollis* Hutch.	Shrub	Mutuzvidzembwa	**Roots:** extract drunk as cough medicine [8]	None found	None found
*Flueggea virosa* (Roxb. ex Willd.) Voigt	Shrub	Mushagahuwe	**Roots:** extract drunk as pneumonia medicine and drunk before sexual intercourse as a contraceptive; dried root powder applied to bitten part as snake antidote and root powder applied on wounds [8]	Contraceptive, pneumonia and snake antidote [1]	Alkaloids, securinine and triterpenes; antifungal, antimalarial, antimicrobial and antioxidant activities [56-59]
*Macaranga capensis* (Baill.) Benth. ex Sim	Tree	Musvosve	**Roots:** extract drunk as aphrodisiac[9]	Male impotence^#^[60]	Antibacterial activity [60]
*Ricinus communis L.	Herb	Mupfuta	**Roots:** teeth washed with root decoction as remedy for toothache [8]. **Seed:** oil applied on sore eyes [8]	Sore eyes [1] and toothache^#^[1,10]	Anti-inflammatory, antiarthritic [61,62], anti-oxidant, antiulcer, antidiabetic, antifertility and antimicrobial [63] properties
*Spirostachys africana* Sond.	Tree	Munhiti	**Roots:** root powder mixed with porridge as remedy for venereal infections [9]	None found	Flavonoids and gallotannins [64], phenolic and antioxidant activity [65]
**Fabaceae***sensu lato*					
*Acacia karroo* Hayne	Tree	Muvunga	**Root:** extract drunk as convulsions remedy [8]; aphrodisiac, gonorrhoea and syphilis [9]	Aphrodisiac, convulsions, gonorrhoea and syphilis [1]	Anti-inflammatory, analgesic [66], antibacterial [67] and antimicrobial [66] activities
*Albizia antunesiana* Harms	Tree	Muriranyenze	**Bark:** extract drunk as constipation remedy [8]. **Leaves:** extract drunk as purgative remedy [8]. **Roots:** extract drunk as aphrodisiac, diarrhoea, gonorrhoea and remedy for infertility in women [8]	Aphrodisiac [1,24], gonorrhoea, infertility in women and as purgative [1]	Anthelmintic activity [15]
*Brachystegia boehmii* Taub.	Tree	Mupfuti	**Bark:** extract drunk as STI medicine [8]	None found	Antibacterial activity [13]
*Cassia abbreviata* Oliv.	Shrub	Muremberembe	**Roots:** extract drunk as abortion, aphrodisiac, constipation, diarrhoea and gonorrhoea medicine [8]	Abortion, aphrodisiac constipation diarrhoea and gonorrhoea [1]	Anthraquinones, triterpenoids [68], antibacterial [55], antimalarial [69] and antiviral [70] activities
*Dalbergia melanoxylon* Guill. & Perr.	Shrub	Mugwiti	**Leaves:** dried leaves smoked as cigarette to treat asthma [9]	Bronchitis^#^ and inflammation in throat^#^[71]	Antimicrobial activity [72]
*Elephantorrhiza goetzei* (Harms) Harms	Shrub	Ntorani	**Roots:** extract drunk as abdominal pains, diarrhoea and gonorrhoea medicine; and mixed with roots of *Piliostigma thonningii* (Schumach.) Milne-Redh. as bilharzia (schistosomiasis) medicine [8]	Abdominal pains [1,24], bilharzias, diarrhoea and gonorrhoea [1]	Anthelmintic activity [15] and stilbenes [73]
*Erythrina abyssinica* Lam. ex DC.	Tree	Mutiti	**Bark:** extract drunk as backache medicine [8]. **Roots:** wounds washed with root extract [8]	Backache and wounds in mouth [1]	Antibacterial [74,75], antifungal [74] and cytotoxic activities [76]
*Indigofera setiflora* Baker	Herb	Ruvavashuro	**Roots:** extract drunk as diarrhoea medicine [9]	None found	None found
*Peltophorum africanum* Sond.	Shrub	Muzeze	**Bark, leaves or root:** extract drunk as syphilis medicine [8]. **Roots:** extract drunk as diarrhoea and STI medicine, root extract used as eye drops for sore eyes and teeth washed with root decoction as remedy for toothache [8]	Diarrhoea and toothache [1], panacea [24] and venereal diseases^#^[1,10]	Antibacterial activity [52,77]
*Piliostigma thonningii* (Schumach.) Milne-Redh.	Tree	Musekesa	**Bark, leaves or root:** extract drunk as cough medicine [8]. **Leaves:**extract drunk as menorrhagia medicine [8]. **Roots:** mixed with roots of *Elephantorrhiza goetzei* as bilharzia (schistosomiasis) medicine [8]	Cough and menorrhagia [1]	Alkaloids, flavonoids, saponins and tannins; antibacterial [16], antimicrobial and antioxidant [78] activities
*Pterocarpus angolensis* DC.	Tree	Mubvamaropa	**Bark:** extract dropped into ear as earache medicine, extract drunk as remedy for menorrhagia [8]. **Roots:** extract drunk as remedy for infertility in women [8]. **Sap:** dropped into sore eyes [8]	Infertility in women and sore eyes [1]; and menorrhagia^#^[1,10]	Antibacterial and cytotoxicity activities [79]
**Flacourtiaceae**					
*Flacourtia indica* (Burm. f.) Merr.	Shrub	Munhunguru	**Leaves:** leaves browsed by mouth as diarrhoea medicine [8]	Diarrhoea [1]	Antibacterial, anti-inflammatory, antimicrobial, antioxidant and antimalarial activities [80]
**Hypoxidaceae**					
*Hypoxis obtusa* Ker Gawl.	Herb	Nhindiri	**Bulb:** bulb chewed and sap swallowed as remedy for abdominal pains [9]	Abdominal pains [1]	Hypoxoside [81] and obtusaside [82]
**Kirkiaceae**					
*Kirkia acuminata* Oliv.	Tree	Mubvumira	**Bark:** extract drunk as diarrhoea, cholera, dysentery and constipation medicine [9]. **Fruits:** fruit juice applied to bitten part as snake antidote and fruit juice applied on wounds [8]	Diarrhoea and wounds [1]	Antibacterial activity [61]
**Lamiaceae**					
*Hoslundia opposita* Vahl	Herb	Hwahwa hwe shiri	**Leaves:** extract dropped into eyes as cataract medicine [8]	Cataract [1]	Alkaloids, flavonoids, saponins, tannins and triterpenes [83,84] and antimicrobial activity [85]
*Leonotis leonurus* (L.) R.Br.	Herb	Mutodzvo	**Leaves:** leaves chewed and sap swallowed as remedy for ulcers [9]	Sores^#^ and haemorrhoids^#^[84]	Anti-inflammatory, cytotoxic and hepatoprotective activities [86]
*Vitex payos* (Lour.) Merr.	Tree	Mutsvubvu	**Leaves:** leaves burnt and smoke inhaled as cough medicine [8,9]	None found	Larvicidal activity [87]
**Loganiaceae**					
*Strychnos cocculoides* Bak.	Tree	Muzumwi	**Roots:** extract drunk as abdominal pains, aphrodisiac, gonorrhoea, infertility in men and sore throat remedy [8]	Abdominal pains and infertility [1]	Antimalarial activity [88]
*Strychnos madagascariensis* Poir.	Tree	Mukwakwa	**Roots:** extract used as eye drops for sore eyes [8]	None found	None found
*Strychnos spinosa* Lam.	Tree	Mutamba	**Roots:** extract drunk as remedy for abdominal pains and gonorrhoea [8]. **Fruits:** extract drunk as remedy for gonorrhoea and genital warts [9]	Abdominal pains [1]	Sterols and triterpenoids [89]
**Malvaceae**					
*Azanza garckeana* (F. Hoffm.) Exell & Hillc.	Tree	Mutohwe	**Roots:** extract dropped into the ear as medicine for earache [8]	Earache^#^[90]	Antimalarial activity [69]
**Meliaceae**					
*Ekebergia benguelensis* Welw. ex C.DC.	Tree	Mudyavarungu	**Bark:** extract drunk as remedy for infertility in men [8]. **Roots:** extract drunk as dysmenorrhea medicine [8]	Dysmenorrhea and infertility in men [1]	Stilbenes [91] and triperpenes [92]
*Entandrophragma caudatum* (Sprague) Sprague	Tree	Mubanana	**Fruits:** burnt fruit peels mixed with vaseline and applied to area affected with genital warts [9]	None found	Limonoids [93]
**Moraceae**					
*Ficus ingens* (Miq.) Miq.	Tree	Mushavhi	**Roots:** extract drunk as cough medicine [8]	Fever^#^[94]	Anti-inflammatory and analgesic properties [95]
*Ficus sur* Forssk.	Tree	Muonde	**Roots:** extract drunk as diarrhoea and syphilis medicine [8]	Diarrhoea in infants [1]	Antibacterial, anti-inflammatory, antimalarial and anti-ulcer activities [96,97]
*Ficus sycomorus* L.	Tree	Muonde	**Roots:** extract drunk as cough medicine [8]	Tuberculosis^#^, cold^#^ and other chest problems^#^[10]	Antibacterial activity [98], alkaloids, saponins and tannins [99]
**Moringaceae**					
*Moringa oleifera Lour	Shrub	Moringa	**Leaves:** extract drunk as diarrhoea medicine [8]. **Roots:** teeth washed with root decoction as remedy for toothache [8]	Digestive disorders [100]	Alkaloids and flavonoids, anti-inflammatory, antioxidant, antimicrobial, antifertility and anticancer [101] activities
**Myrothamnaceae**					
*Myrothamnus flabellifolius* Welw.	Shrub	Rufandichimuka	**Leaves and twigs:** leaves and twigs boiled and drunk as remedy for cold [9]	Cold^#^ and other chest complaints^#^[1,10]	Alkaloids, flavanoids, phenolics, saponins, steroids and tannins [102], antidiabetic [103] and antimicrobial [104] activities
**Myrtaceae**					
*Eucalyptus camaldulensis Dehnh	Tree	Mugamutiri	**Leaves:** extract drunk with *Citrus limon* fruits and *Psidium guajava* L. leaves as cough, flu and fever medicine [8]	Sore throat^#^[105]	Antiproliferative [106] and antimicrobial [107] activities
*Psidium guajava L.	Shrub	Mugwavha	**Leaves:** extract drunk with *Citrus limon* fruits and *Eucalyptus camaldulensis* leaves as cough, flu and fever medicine [8]; infusion of drunk or taken as an enema for diarrhoea [9]	Fever [1]; cough^#^[1,11,85] and diarrhoea^#^[11,85]	Anti-diarrhoeal [108,109], antibacterial [110,111], narcotic [109] and antioxidant [112] properties
*Syzygium cordatum* Hochst. ex C. Krauss	Tree	Mukute	**Bark:** extract drunk as tuberculosis medicine [9]	Cold^#^ and fever^#^[10]	Antibacterial [52] and antifungal [64] activities
**Ochnaceae**					
*Ochna pulchra* Hook.f.	Shrub	Munimu	**Leaves:** leaf sap taken as remedy for stomach problems [9]	Diarrhoea [1]	Antibacterial activity [113]
**Olacaceae**					
*Ximenia americana* L.	Shrub	Mutengeni	**Leaves:** extract drunk as backache medicine [8]	Abdominal pains^#^[114]	Antibacterial [64] and antioxidant [114] activities
*Ximenia caffra* Sond.	Shrub	Munhengeni	**Leaves:** extract drunk as backache medicine [8]. **Roots:** extract drunk as aphrodisiac, diarrhoea, venereal diseases; root powder applied on wounds [8]	Diarrhoea and infertility [1]; and venereal diseases^#^[1,10]	Flavonoids, phenolic and tannins; and antimicrobial activity [115]
**Pedaliaceae**					
*Dicerocaryum zanguebarium* (Klotzsch) Abels	Herb	Ruredzo	**Whole plant:** plant foam inserted into vagina to dilate birth canal [9]	To dilate birth canal [1] and expulsion of placenta^#^[10]	Cytotoxic activity [116]
**Polygalaceae**					
*Securidaca longepedunculata* Fresen.	Shrub	Mufufu	**Roots:** extract drunk as epilepsy medicine and mixed with roots of *Annona stenophylla* and sprinkled around homestead as snake repellent [8]	Epilepsy [1] and snake repellent [24]	Analgesic, anti-inflammatory, hypoglycaemic [117] and antimalarial [88] activities
**Pteridaceae**					
*Pellaea* sp.	Fern	Mudziwebwe	**Leaves and roots:** leaves and roots burnt and smoke inhaled as remedy for chest pains [9]	None found	None found
**Rhamnaceae**					
*Berchemia discolor* (Klotzsch) Hemsl.	Tree	Nyii	**Roots:** extract drunk as abdominal pains medicine [8]	General body pains [1]	Antimicrobial activity [118] and flavonoids [119]
*Ziziphus mucronata* Willd.	Tree	Muchecheni	**Fruits and leaves:** powder applied on boils [8]. **Roots:** extract drunk as abdominal pains, infertility in women medicine and root powder applied on wounds [8]	Skin infections and wounds [1]; body pains^#^ and infertility in women^#^[10]; boils^#^, sores and swellings^#^[11]	Anthelmintic [15] and antimicrobial [120] activities
**Rosaceae**					
*Prunus persica L.	Tree	Mupichisi	**Leaves:** extract drunk as diarrhoea medicine [22]	None found	Antimicrobial, antioxidant [121]; anti-tumour promoter and anti-Oketsu syndrome [122] effects
**Rubiaceae**					
*Crossopteryx febrifuga* (Afzel. ex G. Don) Benth.	Shrub	Mukomberwa	**Bark:** added to porridge as remedy for diarrhoea and dysentery [9]	Diarrhoea^#^ and dysentery^#^[123]	Alkaloids, flavonoids, saponins, steroids, tannins and terpenoids [124], anti-inflammatory and antimicrobial activities [125]
*Vangueria infausta* Burch.	Tree	Mudzvirungombe	**Roots:** extract drunk as diarrhoea medicine [8]	Diarrhoea [1]	Flavonoids, antibacterial [126,127], antimalarial [88] and antifungal [126] activities
**Rutaceae**					
*Citrus limon (L.) Burm. f.	Tree	Mulemoni	**Fruit:** extract of fruit, *Eucalyptus camaldulensis* and *Psidium guajava* leaves drunk as cough, flu and fever medicine [8]	Throat infections^#^ and tonsil^#^[128]	Analgesic, intestinal mucosa protector and antiseptic [128]
**Solanaceae**					
*Nicotiana tobacum L.	Herb	Fodya	**Leaves:** snuff applied on wounds [8]	Wounds [1]	Cytotoxic activity [129] and steroidal glycosides [130]
*Solanum incanum L.	Shrub	Nhundurwa	**Fruits:** child bathed with fruit macerate as remedy for scabies [9]	Rash^#^, ringworm^#^, skin infections^#^ and warts^#^[131]	Saponins; antibacterial, cytotoxicity [16], antioxidant and cytoprotective [132] activities
**Tiliaceae**					
*Corchorus tridens* L.	Herb	Derere	**Roots:** extract drunk as backache medicine [8]	None found	Flavonoids and microbial activity [133]
*Grewia bicolor* Juss.	Shrub	Mutewa	**Roots:** extract drunk as diarrhoea and gonorrhoea medicine [8]	Diarrhoea^#^[10]	Alkaloids, triterpenoids and antibacterial activity [134]
*Grewia flavescens* Juss.	Shrub	Mubhubhunu	**Roots:** extract drunk as menorrhagia medicine [8]	Inducing labour^#^, infertility^#^ and impotence^#^[10]	Triterpenoids [101]
*Grewia monticola* Sond.	Shrub	Mutewa	**Roots:** extract drunk as diarrhoea medicine [8]	Diarrhoea [1]	None found
**Urticaceae**					
*Pouzolzia mixta* Solms	Shrub	Munanzwa	**Roots:** extract instilled into the vagina to dilate birth canal; extract drunk as STI medicine and root powder applied on wounds [8]	To dilate birth canal [1,24] and venereal diseases [1]	Antibacterial activity [52]
**Verbenaceae**					
*Lantana camara L.	Shrub	Mbarambati	**Leaves:** leaf sap applied on body parts infected with ring worm [9]	Eye injuries^#^[10]	Antibacterial [13] and antimalarial [88] activities; flavonoids and triterpenes [135]
*Lippia javanica* (Burm.f.) Spreng.	Shrub	Zimbani	**Leaves and twigs:** boiled leaves and twigs drunk as remedy for cough and cold [9]	Cold and cough [1,10,24]	Antimicrobial activity [136]
**Vitaceae**					
*Ampelocissus africanus* (Lour.) Merr.	Climber	Muzambiringa	**Roots:** extract drunk as diarrhoea medicine [8]	Stomach troubles^#^[137]	Antibacterial activity [138]
*Ampelocissus obtusata* (Welw. ex Baker) Planch.	Climber	Muzambiringa	**Roots:** extract drunk as diarrhoea medicine [8]	Gastro-intestinal complaints^#^[137]	Antibacterial activity [138]
**Zingiberaceae**					
*Zingiber officinale Roscoe	Herb	Tsangamidzi	**Roots:** roots chewed and swallowed as remedy for stomach pains [9]	Digestive disorders^#^[139]	Antimicrobial [16], anti-inflammatory, immuno-modulatory and anti-emetic [140] activities

An asterisk (*) indicates that the taxon is known or believed to be exotic to Zimbabwe and hatch (#) indicates similar use(s) in other countries reported from literature.

**Table 2:** Families with the largest number of medicinal plants (more than 2 species) in south-central Zimbabwe

**Family**	**Number of medicinal plants**	**%**
Fabaceae *sensu lato*	11	11.8
Anacardiaceae	9	9.7
Euphorbiaceae	7	9.5
Asteraceae	4	4.3
Ebenaceae	4	4.3
Tiliaceae	4	4.3
Apocynaceae	3	3.2
Asparagaceae	3	3.2
Lamiaceae	3	3.2
Loganiaceae	3	3.2
Moraceae	3	3.2
Myrtaceae	3	3.2
Meliaceae	2	2.2
Olacaceae	2	2.2
Rhamnaceae	2	2.2
Rubiaceae	2	2.2
Solanaceae	2	2.2
Verbenaceae	2	2.2
Vitaceae	2	2.2

### Growth habit and parts used

**Figure 2:**
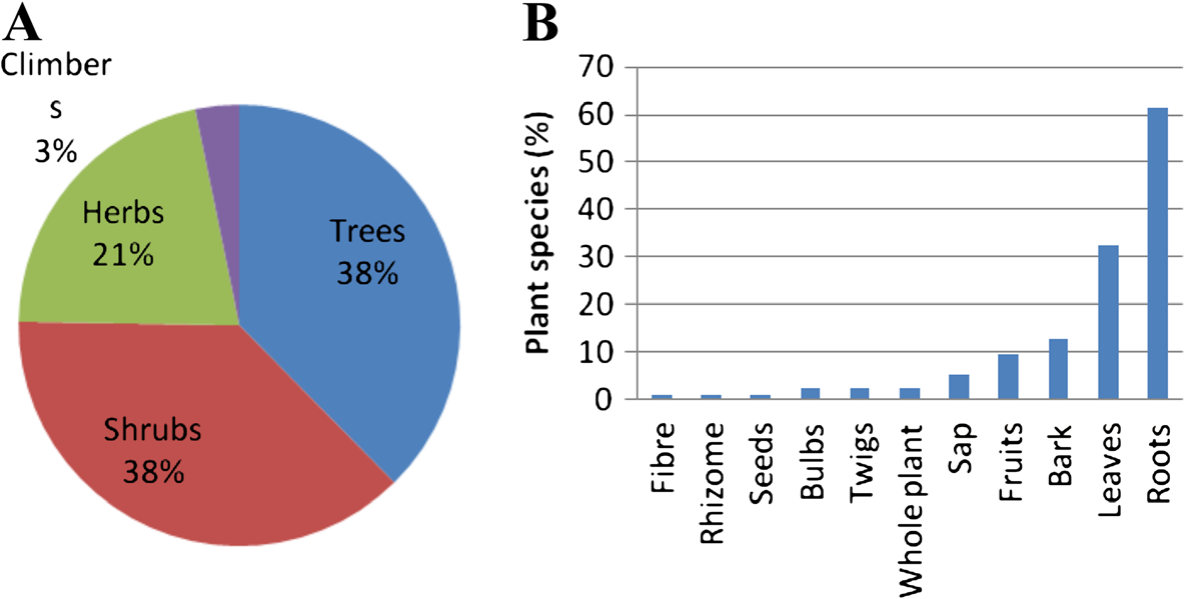
**Characteristics of the plants used as herbal medicines in south-central Zimbabwe.** (**A**) Growth form habit represented in pie diagram and (**B**) plant parts used represented in bar chart.

Trees and shrubs (38% each) are the primary sources of the medicinal plant species in south-central Zimbabwe, followed by herbs (21%) and climbers (3%) (Figure 2A). Extensive use of trees and shrubs in south-central Zimbabwe in preparation of herbal medicines might be linked to their availability throughout the year as they are relatively drought resistant and are not affected by seasonal variations [8]. The plant parts used for making herbal preparations were the bark, bulbs, fibre, fruits, leaves, rhizomes, roots, sap, seeds, twigs and whole plant. The roots were the most frequently used (61.3%), followed by leaves (32.3%), bark (12.9%), fruits (9.7%), sap (5.4%), bulbs, twigs and whole plant (2.2% each), fibre, seeds and rhizomes (1.1% each) (Figure 2B). The use of whole plant as remedy was administered for herbaceous plant species (Table 1). However, harvesting of roots of herbaceous plants for medicinal purposes is not sustainable as it threatens the survival of the same plants used to treat human ailments in south-central Zimbabwe. It is well recognized by conservationists that medicinal plants primarily valued for their root parts and those which are intensively harvested for their bark often tend to be the most threatened by over-exploitation [141].

### Ailments and diseases treated and herbal preparation

**Figure 3:**
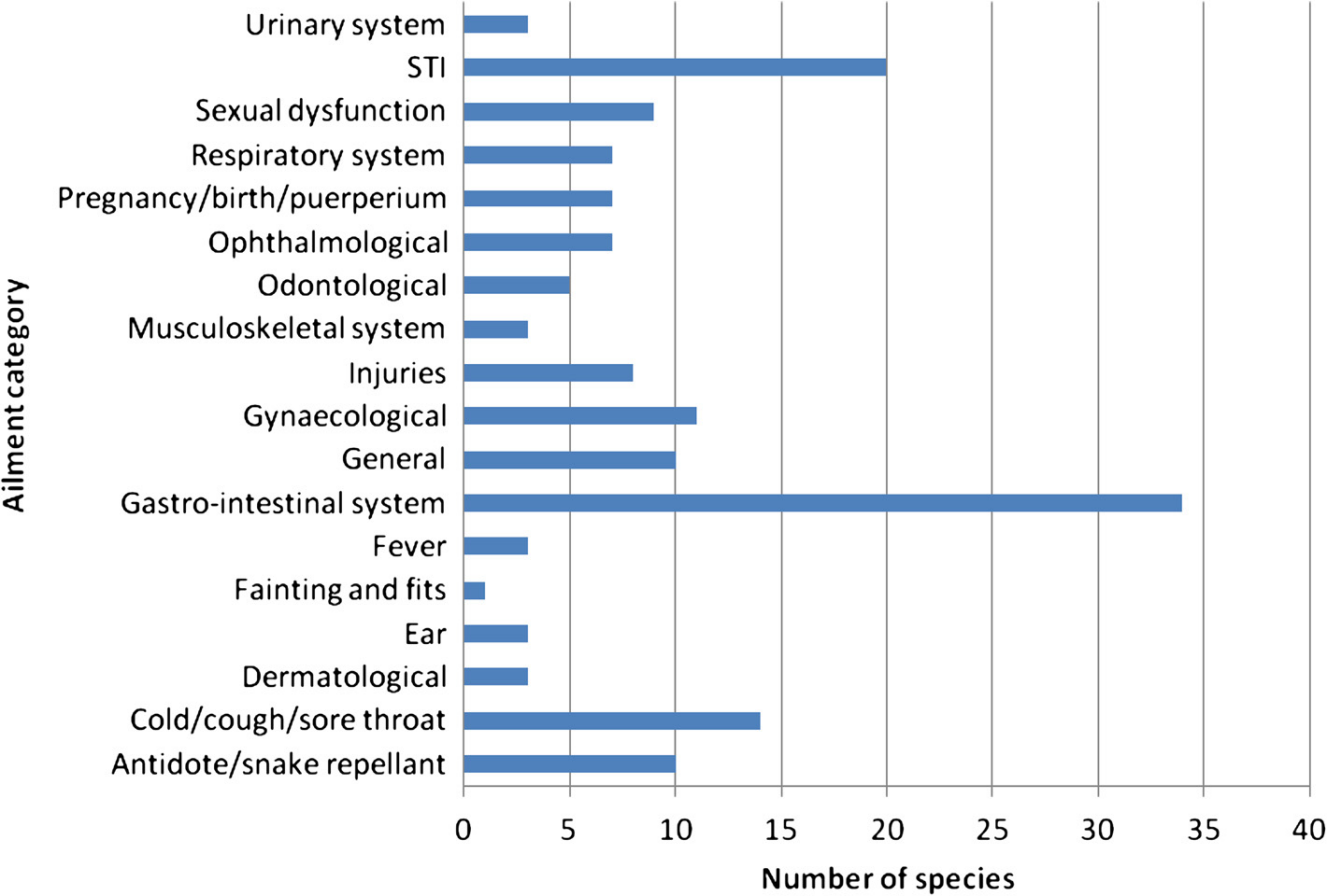
**Major ailments and disease categories and plant species reported.** Most species were reported in more than one ailment category.

The majority of the plant species used (61.3%) had a single therapeutic use, with 19 species (20.4%) used in the treatment of two ailments, 6 species (6.5%) treating three ailments, 5 species (5.4%) treating four ailments, 3 species (3.2%) treating five ailments and *Albizia antunesiana* used to treat six ailments (Table 1). A total of 18 medical conditions were treated using remedies made from medicinal plants (Figure 3). Gastro-intestinal disorders, sexually transmitted infections, cold, cough and sore throat and gynaecological problems were treated with the highest number of medicinal plant species (Figure 3). Gastro-intestinal disorders, particularly cholera, diarrhoea and dysentery are a major concern not only in south-central Zimbabwe but the whole country and; in Mozambique as well, where dysentery and cholera usually result in high mortality rate if not treated promptly [142]. Sexually transmitted infections are a major public health concern in developing countries with their transmission rate regarded as one of the highest in the world [143]. Sexually transmitted infections are one of the most common reasons for people to use herbal medicines and visit traditional healers in Zimbabwe [1,4,8].

Plant remedies were often utilized in the form of extracts (76.3%), sap (11.8%) and powder (6.5%) (Table 1). Other preparation methods included paste, chewing the raw plant and inhaling smoke or vapour generated by burning some of the medicinal plant species. Most of the preparations (69.9%) were prescribed orally in warm water or soft porridge (Table 1). Herbal preparation methods and dosage depend on the type of disease. Some plants were boiled while others were applied directly in fresh form. Some herbal medicines were applied topically, either as bath, massage or lotion. For example, the use of burnt fruit peels of *Entandrophragma caudatum* which were mixed with vaseline and applied on genital areas affected with genital warts [9]. The herbal prescriptions were usually given to patients until patients reported positive results.

Monotherapy preparations made from a single plant species were the most dominant (92.5%); 4.3% and 3.2% of the herbal concoctions were prepared from a combination of two and three species respectively. Those that involved the use of two species included mixing roots of *Annona stenophylla* and *Securidaca longipedunculata* as snake repellent [8]. Roots of *Elephantorrhiza goetzei* and *Piliostigma thonningii* were used as remedy for bilharzia (schistosomiasis) [8]. Water extract of *Eucalyptus camaldulensis* leaves, *Citrus limon* fruits and *Psidium guajava* leaves was taken as a cure for cough, flu and fever [8]. The use of multiple therapies in traditional medicine based on combining plants has recently been shown to increase the efficacy of the herbal medicine [144]. According to Bussmann and Sharon [145], the use of more than one plant species to prepare a remedy for ailments is attributed to the additive or synergistic effects that they could have during ailment treatment.

### Literature based proof of biological and pharmacological activities

Out of 93 medicinal plants used in south-central Zimbabwe, 83 species (89.2%) have proven biological and pharmacological activities (Table 1). The presence of these active ingredients in herbal medicines is directly linked to their ability to prevent or treat ailments. Analysis of the biological and pharmacological activity data (Table 1) shows the wide variety of biological activity of plants used as herbal medicines in south-central Zimbabwe; and the presence of these active ingredients in utilized plants as herbal medicines corroborates the popular traditional knowledge and medicinal uses of the documented plants. The identification of biologically active compounds in herbal medicines needs to be interpreted in the light of the traditional uses of the plants as well as herbal medicine preparation and dosage [146]. It is worth mentioning that most of these activities, mainly the antibacterial, anti-inflammatory, antiparasitic and analgesic properties; and the presence of alkaloids, flavonoids, saponins and tannins confirm the different popular applications of extracts obtained from traditional medicines.

The majority (82.8%) of the 93 plant species used as traditional medicines in south-central Zimbabwe have similar applications in other regions of Zimbabwe as well as other parts of the world (Table 1). The medicinal uses of 49 species (52.7%) are supported by reports of similar uses elsewhere in Zimbabwe and 40 species (43%) have similar uses in the other parts of the world (Table 1). Such similarities in the cross-cultural usage of the traditional plant remedies are a strong indication of the bioactivity potential of the documented plant species. The following 16 medicinal species (17.2%) appear not to be popular for the ethno medicinal uses documented in Zimbabwe: *Androstachys johnsonii* (aphrodisiac), *Brachystegia boehmii* (sexually transmitted infections), *Bridelia mollis* (cough), *Carissa bispinosa* (cough and diarrhoea), *Coleochloa setifera* (pneumonia), *Commiphora marlothii* (sexually transmitted infections), *Corchorus tridens* (backache), *Entandrophragma caudatum* (genital warts), *Indigofera setiflora* (diarrhoea), *Pellaea* sp. (chest pains), *Prunus persica* (diarrhoea), *Searsia dentata* (ulcers, diarrhoea and stomach problems), *Searsia pyroides* (cough), *Spirostachys africana* (venereal infections), *Strychnos madagascariensis* (sore eyes) and *Vitex payos* (cough) (Table 1). These findings of new ethno medicinal plant uses in south-central Zimbabwe shows the importance of the documentation of such traditional indigenous knowledge. Some of these species can therefore be targeted for phytochemical and pharmacological studies with the aim of identifying active ingredients contained by such plants resulting in them having unique therapeutic uses.

This review showed substantial commonality in the general use of medicinal plants in south-central Zimbabwe, the other regions of Zimbabwe and the rest of the world. For example, ten plant species used to treat at least four ailments in south-central Zimbabwe include *Albizia antunesiana* (six ailments), *Annona stenophylla* (four ailments), *Cassia abbreviata* (five ailments), *Elephantorrhiza goetzei* (four ailments), *Flueggea virosa* (four ailments), *Kirkia acuminata* (five ailments), *Peltophorum africanum* (four ailments), *Pterocarpus angolensis* (four ailments), *Sansevieria hyacinthoides* (five ailments), *Strychnos cocculoides* (five ailments) and *Ziziphus mucronata* (four ailments) (Table 1). With the exception of *Sansevieria hyacinthoides*, the other nine species have been documented by Gelfand et al. [1] as valuable medicinal plants in most regions of Zimbabwe with at least six medicinal applications each. Literature search showed that the roots of *Albizia antunesiana* are widely used in tropical Africa to treat abdominal pains, cuts, depressed fontanelle in infants, gonorrhoea and other sexually transmitted diseases, infertility in women, painful and swollen legs, pneumonia, prevent abortion, sore eyes, sore throat, tonsillitis, tuberculosis and ulcers [147]. A bark infusion of *Albizia antunesiana* is taken to treat constipation and applied externally to cuts; whereas crushed leaves are used as an enema for their purgative action and as a dressing to treat oedema of the legs [147]. Previous research by Gelfand et al. [1] showed wide use of *Annona stenophylla* in traditional medicine in Zimbabwe. Infusion of *Annona stenophylla* root or bark is used to treat abdominal pains, boils, chest pains, constipation, diarrhoea, dysmenorrhea, hiccoughs, oedema, sexually transmitted diseases and sprains [1]. All plant parts of *Cassia abbreviata* are used in tropical Africa to treat gastro-intestinal disorders, bilharzia, diarrhoea, dysmenorrhea, eye problems, haematuria, headache, malaria, pneumonia, snakebites, toothache and venereal diseases [68]. Decoction of all plant parts of *Cassia abbreviata* are used as aphrodisiac, abortifacient, purgative, tonic and vermifuge [68]. Root infusion of *Elephantorrhiza goetzei* is widely used in Zimbabwe as remedy for abdominal pains, backache, bilharzia, constipation, depressed fontenelle, diarrhoea and gonorrhea [1]. *Flueggea virosa* is an important medicinal plant in tropical Africa, used for the treatment of a wide variety of ailments, alone or in combination with other plants. All plant parts of *Flueggea virosa* are used to treat frigidity, liver, bile, kidney, testicular inflammation, sterility, urinary and venereal diseases [56]. All plant parts of *Kirkia acuminata* are used in traditional medicine in Zimbabwe as herbal medicine for abdominal pains, antidote, cough, emetic and wounds [1]. The bark and root extracts of *Peltophorum africanum* are traditionally used in southern Africa to treat acute and chronic pains, boosting resistance to diseases, depression, diarrhoea, dysentery, infertility, intestinal parasites and wounds [84]. The bark of *Pterocarpus angolensis* is widely used in tropical Africa as an astringent to treat diarrhoea, heavy menstruation, nose bleeding, headache, stomachache, schistosomiasis, sores and skin problems [148]. Leaves, rhizomes and roots of *Sansevieria hyacinthoides* are widely used in tropical Africa to treat ear infections, haemorrhoids, intestinal worms, measles, prevention of miscarriage, sexually transmitted infections, stomach disorders, toothache and ulcers [31]. All plant parts of *Strychnos cocculoides* are widely used in Zimbabwe to treat abdominal pains, amenorrhoea, cough, diarrhoea, gonorrhea, hydrocele, infertility, sore eyes and sore throat [1]. Medicines obtained from infusion of the roots, bark, leaves and/or fruits of *Ziziphus mucronata* are used to treat bilharzia, boils, chronic cough, depressed fontanelle, diarrhoea, dysmenorrhoea, infertility in women, menorrhagia, oedema, pneumonia, snake bite, toothache, venereal diseases and wounds [1].

### Future research and perspectives

This review showed that local people in south-central Zimbabwe rely on traditional medicines to treat a wide spectrum of human ailments and are knowledgeable about the identities and applications of medicinal plants. Many people in south-central Zimbabwe are still dependent on medicinal plants, at least for the treatment of basic human ailments like cold, cough, diarrhoea, fever, skin infections, sexually transmitted infections, sore eyes and tooth infections. Data collected in the present review illustrates that gastro-intestinal disorders and sexually transmitted infections are treated with the highest number of medicinal plant species. These findings correlate strongly with observations made by Ribeiro et al. [142] that cholera, diarrhoea and dysentery are a major concern in Mozambique and southern Africa as well as findings made by Van Vuuren and Naidoo [143] that sexually transmitted infections are a major public health concern in developing countries. Reports of similar medicinal applications of the documented plants in south-central Zimbabwe, other regions of Zimbabwe and the rest of the world indicate that these species are valuable sources of ethnomedicines. This comparative analysis strengthens the firm belief that traditional indigenous knowledge represent not only an important heritage, developed over the centuries, but also considerable mass of data that should be exploited in order to provide new and useful knowledge on plant resources. It is therefore, necessary to preserve this indigenous knowledge on traditional medicines by proper documentation, identification of plant species used, herbal preparation and dosage. This inventory will assist future workers on the selection of herbal plants to evaluate for phytochemical safety and pharmaceutical efficacy. There is also need for more research on the active compounds of these herbal medicines, some of which have already shown interesting biological and pharmacological activities as shown in Table 1. There is need to establish the link between the biological activity and particular compounds responsible for the wide use of these medicinal plants. The documented indigenous knowledge in south-central Zimbabwe and available scientific literature strongly suggests that at least some of the plants used as herbal medicines can be potential sources of newer drugs.

At the present moment, phytochemistry and pharmacological analysis of traditional medicines occupy a key position in medicinal plant research and indigenous knowledge systems. Sharing of such knowledge is crucial for maintaining options for the use of traditional medicines, particularly as use of alternative medicine is growing because of its moderate costs and increasing faith in herbal medicines. Significant levels of global knowledge on conventional pharmaceuticals originated from indigenous traditional knowledge. For example, many of the conventional drugs available on the market today have a long history of use as traditional medicines, among them are aspirin, opium and quinine. While south-central Zimbabwe is endowed with a strong culture of herbal medicine usage for primary health care, there is need to standardize the drug preparation, dosage and route of administration. Validating the correlations of the ethno medicinal uses, bioactive substances, biological and pharmacological effects is of special importance and is still the primary task for future research. Efforts are also needed to investigate the physiological and biochemical functions demonstrated by these species, identifying the individual bioactive natural products and illustrate their mechanisms of action.

Like most African countries, Zimbabwe is an important repository of medicinal plants usage in primary healthcare. This is reflected in the great diversity of plants used for medicinal purposes in south-central Zimbabwe as well as in the wide range of their applications and associated traditional medicine procedures. There is a growing upsurge in demand for traditional medicines in Zimbabwe for various human ailments. As demand for medicinal plants continue to accelerate, awareness creation should be made among local communities to ensure sustainable use and conservation of the medicinal plants. A collaborative approach for sustainable use, conservation and management of medicinal plants should be put into place and involve all stakeholders. Communities in south-central Zimbabwe should be actively involved in plant resource management as they depend on these natural resources for their primary healthcare needs. It is hoped that this will strike a balance between meeting their health needs and wise use of plant resources to ensure sustainable development. The most serious threats to medicinal plants, like any other forms of biodiversity are habitat loss and fragmentation, climate change and invasive species. It is not known whether over-exploitation of medicinal plants is an issue in south-central Zimbabwe. However, future studies in south-central Zimbabwe should focus on how local communities use and manage medicinal plants. Such studies will help in understanding how local communities relate to the plant resources that they use as medicines.

## Competing interests

The author declares that he has no competing interests.

## Authors’ contributions

AM conceptualized the study and wrote the manuscript. The author read and approved the final manuscript.
